# Rosai-Dorfman disease with pulmonary involvement mimicking bronchogenic carcinoma

**DOI:** 10.1186/s13019-020-1085-6

**Published:** 2020-02-21

**Authors:** Haneen Al-Maghrabi, Ahmed Elmahrouk, Maun Feteih, Ahmed Jamjoom, Jaudah Al-Maghrabi

**Affiliations:** 1 Department of Pathology, King Faisal Specialist Hospital and Research Center, MBC-J16, P.O. Box 40047, Jeddah, 21499 Saudi Arabia; 2 Department of Cardiothoracic Surgery, King Faisal Specialist Hospital and Research Center, Jeddah, Saudi Arabia; 3 Department of Medicine, Pulmonary medicine Unit, King Faisal Specialist Hospital and Research Center, Jeddah, Saudi Arabia; 4 Department of Pathology, King Abdulaziz University, Jeddah, Saudi Arabia

**Keywords:** Rosai-Dorfman disease, Bronchial mass, Brochogenic carcinoma

## Abstract

**Background:**

Rosai-Dorfman disease is a histiocytic lesion that affects lung rarely.

**Case presentation:**

We present a 52-year-old female diagnosed with right intrabronchial mass invading the bronchial wall and the extrabronchial tissues with lymphadenopathy. Multiple bronchoscopic biopsies were not diagnostic. Pneumonectomy was performed and postoperative histology revealed marked mucin impaction and bronchial dilatation. The pulmonary tissue showed areas of hemorrhage and chronic inflammation. The mass exhibited an excessive number of lymphocytes, plasma cells, and numerous histiocytes engulfing them (lymphocytophagocytosis). These histiocytes were S100 protein and CD68 positive. These features are consistent with Rosai-Dorfman disease.

**Conclusion:**

Rosai-Dorfman Disease with pulmonary affection can be misdiagnosed as malignancy. Careful histological examination of the specimen for emperipolesis or lymphocytophagocytosis together with S100 protein and CD68 positivity are the clue for proper diagnosis.

## Background

Rosai-Dorfman disease (RDD) is a histiocytic lesion that commonly occurs in young males [1]. Typically, it presents as non-tender bilateral cervical lymphadenopathy [2] and extra-nodal affection is rare [3]. The exact etiology is not clear but infectious or autoimmune mechanisms were suggested [4]. Pulmonary presentation of the disease and its management is rarely reported.

## Case presentation

**Fig. 1:**
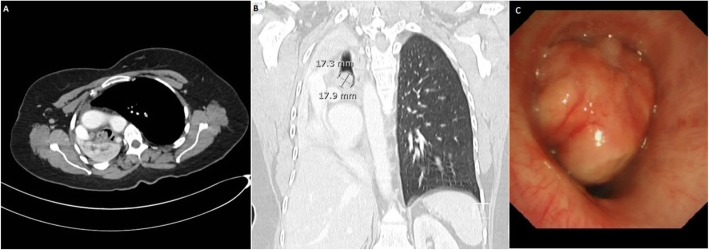
**a** and **b** Preoperative CT chest showing a right bronchial mass in the right main bronchus with total lung collapse and ipsilateral mediastinal shift. **c**: Bronchoscopy showing the mass occluding the right main bronchus

**Fig. 2:**
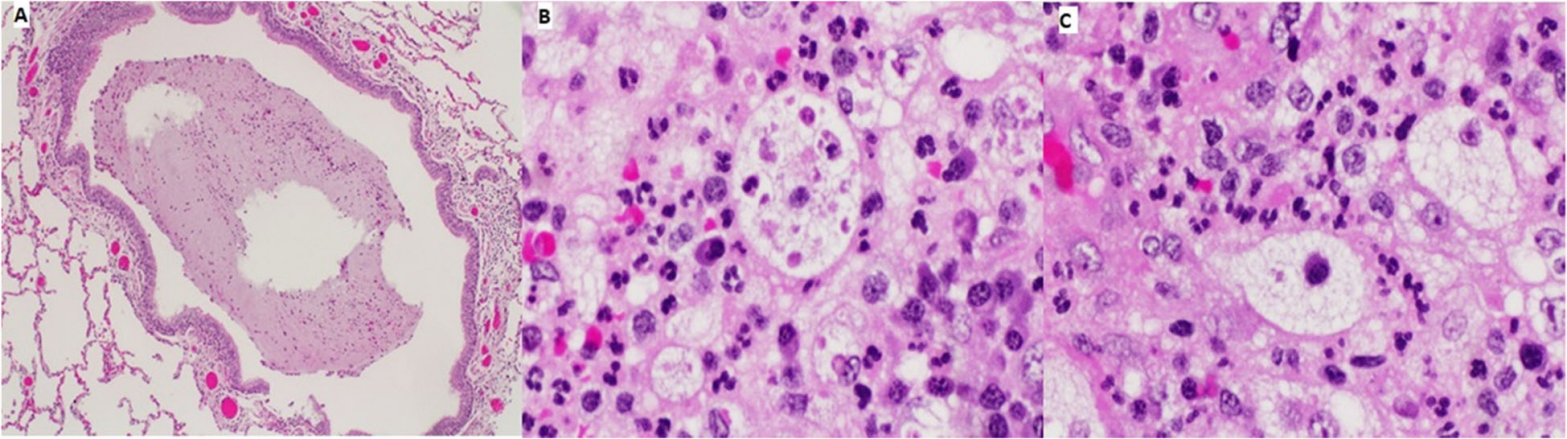
**a**: Lung parenchymal tissue with marked bronchiole dilatation filled with mucin plugs (H&E; 4x); **b**: High-power view showing lymphocytophagocytosis by histiocytes (H&E; 40x); **c**: High-power view showing histiocyte engulfing plasma cell (H&E; 40x)

**Fig. 3:**
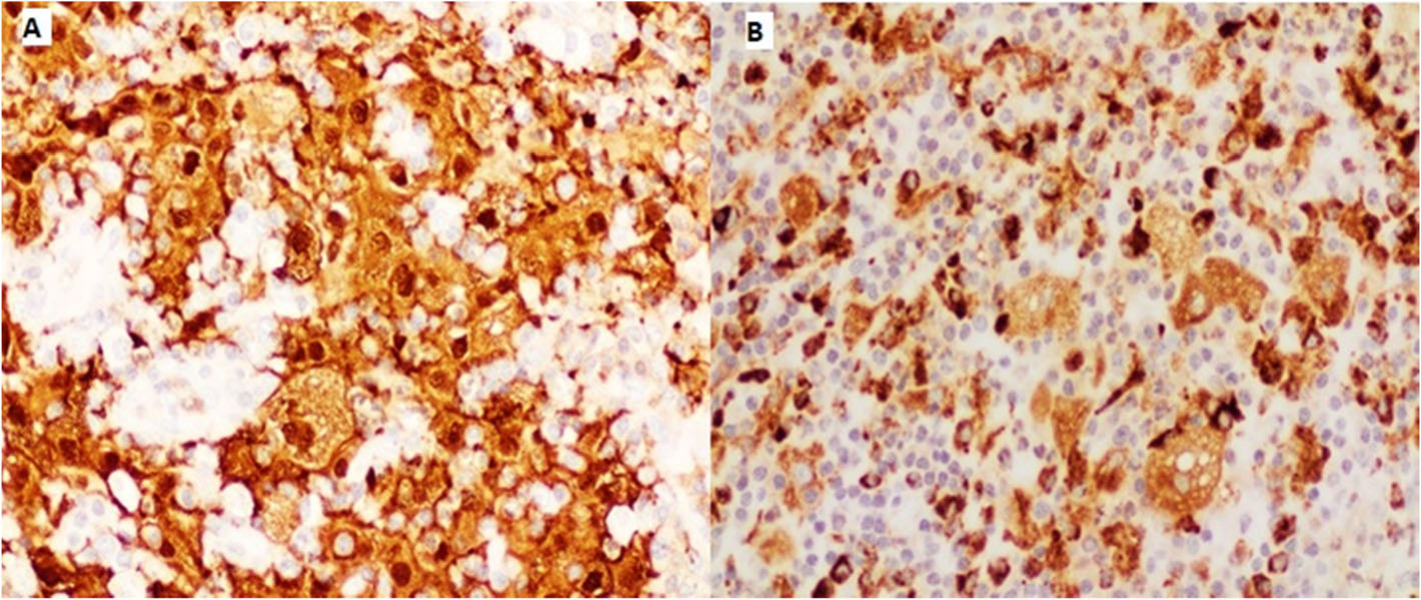
**a**: large histiocytes are diffusely positive for S100 (20x); **b**: Histiocytes diffusely positive for CD68 (20x)

**Fig. 4:**
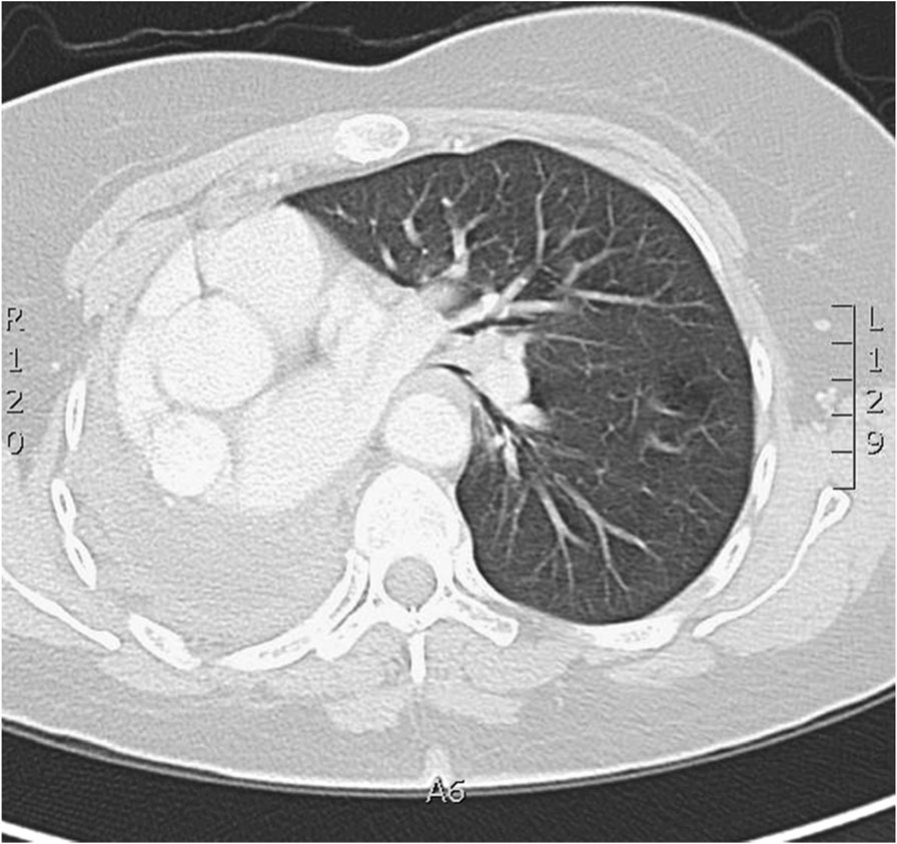
Follow up CT chest after 6 months shows Right-sided pneumectomy with slightly hyperinflated contralateral lung

A 52-year-old female with a history of hypothyroidism presented with continuous cough and shortness of breath. General examination was normal and local chest examination revealed absent breathing sounds on the right side. Pulmonary function test showed a mixed obstructive and restrictive lung pathology. Chest x-ray revealed complete opacity on the right side and contralateral mediastinal shift. Computed tomography (CT) scan of the chest showed infiltrative right hilar soft tissue lesion measures 4 × 5 cm with an endobronchial extension of the lesion obliterating the right main and upper lobe bronchus (Fig. 1a and b). Bronchoscopy showed a large mass occluding the right main bronchus and encroaching on the carina (Fig. 1c). Multiple biopsies were taken but were not diagnostic. After a multidisciplinary meeting between pulmonary, radiology and cardiothoracic surgery teams, a decision was made to proceed for surgical intervention. Although, lobectomy including excision of the bronchial involvement was deemed feasible, intra-operatively, preservation of a healthy and functioning lobe was not possible and pneumonectomy was done. Initial examination of the lung specimen on the operating table showed a large mass at the right main bronchus encroaching on the carina with bronchial wall invasion extending to the extra-nodal tissue. There were multiple lymph nodes enlargement and were biopsied. Gross examination showed the main bronchus filled with mucus plug and serial sectioning revealed variable sized bronchioles filled with thick mucoid secretions. Microscopic examination showed lung parenchymal tissue with marked mucin impaction that plugged the bronchial tree and associated with bronchial dilatation (Fig. 2a). The adjacent pulmonary tissue showed areas of hemorrhage, lung collapse and chronic inflammation. No evidence of malignancy was seen. However, tissue fragments from the peribronchial mass exhibits an excessive numbers of lymphocytes, plasma cells, some neutrophils and most notably numerous histiocytes with abundant foamy cytoplasm and large vesicular nucleus. Many of these histiocytes engulfed numerous intact lymphocytes and plasma cells within their cytoplasm (emperipolesis or lymphocytophagocytosis) (Fig. 2b and c). These large histiocytes were positive for S100 protein (Fig. 3a) and Cluster of Differentiation 68 (CD68) (Fig. 3b) and were negative for Cluster of Differentiation 1a (CD1a) and Langerin. Kappa and lambda stains did not show obvious light chain restriction and Immunoglobulin G 4/ Immunoglobulin G IgG4/ IgG was less than 5%. Special stains for Fungi, Gomori Methenamine-Silver Nitrate Stain (GMS) and Periodic acid–Schiff (PAS) were negative. The histopathological and immunohistochemical features were consistent with Rosai-Dorfman disease. After 6 months follow-up, the patient was asymptomatic, and no tumor recurrence or metastasis was detected (Fig. 4).

## Discussion

Rosai-Dorfman disease is an uncommon nonmalignant histiocytic proliferative disorder that was originally described as a discrete pathological disorder in 1969 by Rosai and Dorfman [1]. Human herpesvirus-6 (HHV-6) and parvovirus B19 were the most common leading cause of infection and dysregulation in histiocytic proliferation [2]. Moreover, several reports stated that HHV-6 infection found in histiocytes, while parvovirus B19 and Epstein-Barr virus (EBV) antigens targeted lymphocytes, engulfed by the proliferating histiocytes [5]. Other studies failed to find any association between HHV-6, parvovirus B19 and RDD which is consistent with our findings [2]. The mechanism of histiocytic proliferation in infectious etiology is not well known. Some authors suggested that macrophage colony-stimulating factor (M-CSF) stimulates macrophages which lead to macrophages/ histiocytic proliferation [4, 6]. On the other hand, autoimmune reaction can cause an acquired internal dysregulation which results in disturbance of cellular apoptotic signaling pathway mechanism that lead to macrophages/ histiocytic proliferation [5]. Some studies suggest an association between RDD with pulmonary affection and IgG4 related diseases [2]. However, no strong clinical or genetic evidence supported this and IgG4 disease was excluded in our patient.

Rosai-Dorfman disease involving the tracheobronchial tree is rarely reported in the literature [7]. The disease can involve larynx, subglottic area, trachea, and bronchi. It can cause an exophytic mass either intraluminal or intramural. Patients usually present with symptoms related to bronchial compression [7]. Our case presented with the unique finding in radiology examination mimicking primary carcinoma of the lung due to massive mucin impaction within the main bronchus and its dilated branches. Repeated biopsies were negative which indicates that sufficient tissue is necessary for proper histological examination. Pathological examination is the gold standard diagnosis which demonstrates a marked increase in histiocytes engulfing in their cytoplasm numerous intact lymphocytes, plasma cells, and sometimes erythrocytes, a feature well-known as emperipolesis [1]. The differential diagnosis include benign lymphoid hyperplasia with sinus histiocytosis which will lack the emperipolesis seen in RDD, Langerhans cell histiocytosis in which the cells are positive for S100, langerin, and CD1a. Other differentials include leprosy infection, rhinoscleroma, melanoma, and metastatic carcinoma [1, 4].

The prognosis of RDD is generally benign. Many cases of RDD undergo spontaneous complete resolution, especially in cases with nodal involvement only [4]; while other cases have an unpredictable outcome. In severe cases of RDD particularly with extra-nodal involvement, or cases with vital organs compression such as kidney and CNS, the patient might have worse outcome due to physiological and immunological complications. In cases with nodal involvement and spontaneous remission, observation is the usual modality of treatment. Medical and/ or surgical treatment is needed when there is extra-nodal involvement with obstruction or compression symptoms [2, 4]. Rosai-Dorfman disease may not respond completely to therapy, although chemotherapy was effective in some cases [8]. Some authors suggested the use of low-dose corticosteroids, methotrexate, or 6-mercaptopurine in cases of extra-nodal involvement. The recurrence rate in RDD involving tracheobronchial airway had been reported, therefore long term follow up is recommended [7].

## Conclusion

In conclusion, Rosai-Dorfman disease with pulmonary affection can be misdiagnosed as malignancy. Careful histological examination of the specimen for emperipolesis or lymphocytophagocytosis together with S100 protein and CD68 positivity are the clue for proper diagnosis.

## Data Availability

Data are available on request to the corresponding author.

## Abbreviations

CD1a: Cluster of Differentiation 1a
CD68): Cluster of Differentiation 68
CT: Computed tomography
EBV: Epstein-Barr virus
GMS: Gomori Methenamine-Silver Nitrate Stain
HHV-6: Human herpesvirus-6
IgG: Immunoglobulin G
M-CSF: Macrophage colony-stimulating factor
PAS: Periodic acid–Schiff
RDD: Rosai-Dorfman disease
